# The addition of a polyglutamate domain to the angiogenic QK peptide improves peptide coupling to bone graft materials leading to enhanced endothelial cell activation

**DOI:** 10.1371/journal.pone.0213592

**Published:** 2019-03-11

**Authors:** Nicholas W. Pensa, Andrew S. Curry, Michael S. Reddy, Susan L. Bellis

**Affiliations:** 1 Department of Biomedical Engineering, University of Alabama at Birmingham, Birmingham, Alabama, United States of America; 2 School of Dentistry, University of California, San Francisco, California, United States of America; 3 Department of Cell, Developmental, and Integrative Biology, University of Alabama at Birmingham, Birmingham, Alabama, United States of America; Universite de Technologie de Compiegne, FRANCE

## Abstract

Vascularization of bone grafts is vital for graft integration and bone repair, however non-autologous graft sources have limited potential to induce angiogenesis. Accordingly, intensive research has focused on functionalizing non-autologous materials with angiogenic factors. In the current study we evaluated a method for coupling an angiogenic peptide to the surface of two clinically-relevant graft materials, anorganic bovine bone (ABB) and synthetic hydroxyapatite (HA). Specifically, the VEGF-derived “QK” peptide was synthesized with a heptaglutamate (E7) domain, a motif that has strong affinity for calcium phosphate graft materials. Compared with unmodified QK, a 4–6 fold enrichment was observed in the binding of E7-modified QK (E7-QK) to ABB and HA. The E7-QK peptide was then assessed for its capacity to stimulate angiogenic cell behaviors. Human umbilical vein endothelial cells (HUVECs) were treated with solutions of either QK or E7-QK, and it was found that QK and E7-QK elicited equivalent levels of cell migration, tubule formation and activation of the Akt and ERK signaling pathways. These data confirmed that the inherent bioactivity of the QK sequence was not diminished by the addition of the E7 domain. We further verified that the activity of E7-QK was retained following peptide binding to the graft surface. HA disks were coated with QK or E7-QK, and then HUVECs were seeded onto the disks. Consistent with the increased amount of E7-QK bound to HA, relative to QK, markedly greater activation of Akt and ERK 1/2 was observed in cells exposed to the E7-QK-coated disks. Taken together, these results suggest that the E7 domain can be leveraged to concentrate angiogenic peptides on graft materials, facilitating delivery of higher peptide concentrations within the graft site. The ability to endow diverse graft materials with angiogenic potential holds promise for augmenting the regenerative capacity of non-autologous bone grafts.

## Introduction

More than 2 million bone grafting procedures are performed each year world-wide [1]. Autologous bone is the ideal graft material for these procedures as it retains the osteoinductive growth factors and cells important for effective graft incorporation. However, autologous bone grafts have a number of disadvantages including the risk of secondary surgery site morbidity, as well as the finite amount of donor bone available [2, 3]. To address these issues, non-autogenous graft materials including allograft, xenograft, and synthetic substrates are commonly used as alternatives [4]. These materials are abundant, however, they often lack the critical osteoinductive factors necessary for stimulating graft integration into the surrounding tissue [5]. Without these factors, the potential for complete bone repair is diminished.

Multiple strategies have been pursued to improve the osteoregenerative potential of non-autogenous grafts. One approach is to passively coat the grafts with growth factors that enhance new bone formation such as BMP2, VEGF, PDGF, and FGF [6–12]. However, passively adsorbed growth factors are typically weakly bound to the graft surface, and are therefore rapidly released following graft implantation. This poses several problems. First, inadequate growth factor binding to the graft precludes sustained delivery of growth factors within the graft site, and secondly, supraphysiologic doses of growth factors are usually required to compensate for the rapid bolus release [7, 13, 14]. Furthermore, the dissemination of high concentrations of growth factors outside of the graft site can cause deleterious side effects. For example, systemic release of recombinant BMP2 (rBMP2) induces inflammation and ectopic calcification [13, 15], whereas high dose rVEGF dissemination can cause increased vascular permeability [16]. For these reasons, improved methods are needed for coupling osteoregenerative factors to graft materials, enabling more controlled and localized delivery.

One promising method for functionalizing graft materials with bioactive factors involves the use of polyglutamate or polyaspartate sequences as binding domains for hydroxyapatite (HA), a calcium phosphate crystal that comprises the principal constituent of bone mineral. These negatively-charged domains, consisting of either repeating glutamate or aspartate residues, bind through ionic interactions with the Ca^2+^ present in HA [17, 18]. Polyglutamate and polyaspartate motifs are found within endogenous bone-resident proteins such as bone sialoprotein and osteocalcin, and their natural function is to localize these proteins to bone matrix [17–20]. To mimic this process, polyglutamate sequences have been incorporated into synthetic bioactive peptides to improve peptide binding to a variety of graft materials including allograft, anorganic bovine bone (ABB), and synthetic HA [21–27]. As an example, our group determined that adding a heptaglutamate (E7) domain to an osteoinductive BMP2-derived peptide (BMP2pep) significantly increased the amount of peptide that could be loaded onto the graft, as well as retention of the peptide on the graft following implantation [21]. In addition, grafts coated with E7-modified BMP2pep elicited significantly more new bone formation than grafts passively adsorbed with unmodified BMP2pep in a rat mandibular defect model [21]. These results confirmed that better coupling of osteoinductive factors to the graft surface was effective in enhancing the bone regenerative response.

Polyglutamate domains have been primarily used to couple osteoinductive and cell adhesive peptides to graft materials [21, 22, 25, 26], however angiogenic peptides hold considerable potential for augmenting osteogenesis. Angiogenesis plays a crucial role in bone healing [28, 29], and the lack of rapid vascularization into a graft site is one of the major barriers hindering bone regeneration [30]. One of most potent inducers of angiogenesis is VEGFA. VEGFA stimulates the migration and proliferation of endothelial cells through its activation of surface receptors such as VEGFR2 (KDR) [31]. A wealth of studies has established that VEGFA promotes neovascularization within injured tissues [32], and also enhances graft integration and viability [7, 33, 34].

Given the importance of neovascularization in osteoregeneration, the current investigation aimed to functionalize graft materials with an angiogenic peptide derived from VEGFA, referred to as the “QK” peptide [35]. The QK peptide encompasses amino acids 17–25 of the VEGFA protein, a sequence that constitutes the principal domain within VEGFA that binds VEGFR2 [36]. As with VEGFA, the binding of QK to endothelial cell receptors stimulates signaling events, such as ERK and Akt activation, that promote angiogenic behaviors including cell migration and *in vitro* tubule formation [35, 37]. Moreover, *in vivo* studies have confirmed QK’s capacity to induce angiogenesis in a number of animal models [37–39]. In view of these findings, we investigated whether synthesizing QK with an E7 domain would increase peptide association with calcium phosphate graft materials, thereby facilitating more efficient peptide delivery within graft sites. Here we report that E7-modified QK peptides (E7-QK) exhibited significantly better binding than unmodified QK to two types of graft materials, ABB and synthetic HA. Importantly, the increased concentration of E7-QK vs. QK on the graft surface elicited more robust activation of endothelial cells seeded onto the grafts. Collectively these studies highlight the use of E7-QK peptides as a promising therapeutic modality for improving vessel in-growth into bone graft sites.

## Materials and methods

### VEGF mimetic peptides

All peptides utilized in this study were custom synthesized by Bachem. The QK peptide (KLTWQELQLKYKGI) was synthesized with or without an E7 domain, along with a three-glycine linker sequence to separate the QK domain from the E7 moiety. More specifically, the E7-QK peptide sequence is KLTWQELQLKYKGIGGGEEEEEEE, and the QK sequence is KLTWQELQLKYKGIGGG. For some experiments, E7-QK and QK peptides were also modified with a fluorescein isothiocyanate (FITC) group to facilitate studies of peptide binding to graft. The FITC tag was chemically conjugated to the N-termini of the peptides. Peptides used for cell signaling studies did not have the FITC tag. Lyophilized peptides were reconstituted in deionized water at a concentration of 1 mg/mL, aliquoted, and stored in -20°C until use. rVEGF (R&D Systems, 293-VE-010) was reconstituted to a 5 μg/mL stock solution, and stored at -20°C.

### Graft materials

0.4 g of HA powder (MP Biomedicals, 02150162) were pressed into disks using a 15.875 mm die under 3000 psi as in our prior publications [40]. The HA disks were then sintered at 1000°C in a Thermolyne 48000 series furnace for 4 hrs and allowed to return gradually back to room temperature. Anorganic Bovine Bone (ABB, BioOss) was purchased from Geistlich. ABB graft and HA disks were stored under sterile dry conditions and autoclaved before use.

### Endothelial cell culture

Human Umbilical Vein Endothelial Cells (HUVECs) were purchased from ATCC (HUV-EC-C CRL-1730) and cultured in F-12K media (ATCC 30–2004) with 10% Fetal Bovine Serum (FBS), 0.1 mg/mL heparin (Sigma H3393), 1% antibiotic/antimycotic supplement (Invitrogen), and endothelial cell growth supplement (ECGS, Sigma E0760). Prior to experiments, cells were incubated for 12 hrs in serum-free F-12K media. Cell passages 3–9 were used for all experiments.

### Binding of FITC-labeled peptides to bone graft materials

FITC-labeled QK and E7-QK peptides were used to monitor peptide binding to graft materials. Stock solutions of QK or E7-QK were diluted to a final concentration of 1 μM in Tris-buffered saline (TBS). These solutions were used to coat HA disks or 25 mg of ABB for time points ranging from 30 min to 6 hrs. After coating, samples were briefly washed with TBS to remove any unbound peptide and then imaged using a Leica MZ16F fluorescent dissecting microscope. Grafts coated with either QK or E7-QK, along with uncoated controls, were imaged in the same field to enable a direct comparison. Images were captured using a Hamatsu camera system and SimplePCI imaging software. Pixel intensity for the captured images was evaluated using ImageJ software to measure differences in FITC-labeled peptides bound to graft substrate.

### Endothelial cell migration

A linear scratch defect model was used to monitor the migration of endothelial cells. HUVECs were seeded at a density of 1x10^5^ cells/well in a 48 well plate, and allowed to grow to confluency. A linear scratch approximately 600 μm wide was introduced into the monolayer, and cells were then incubated at 37°C in serum-free F-12K media containing either 50 ng/mL of rVEGF, or 25 nM of either QK or E7-QK peptide. The scratch wound cultures were incubated in the EVOS FL Auto Cell Imaging System (ThermoFisher Scientific) at 37°C, and images were taken at 6 and 12 hrs. Relative closure of the scratch wound was quantified using EVOS FL Auto Cell Imaging System Software.

### Endothelial tubule formation

150 μL/well of Geltrex^™^ LDEV-Free Reduced Growth Factor Basement Membrane Matrix (ThermoFisher Scientific, A1413202) substrate was placed inside a 24 well plate and incubated for 30 min at 37°C to solidify the matrix. Prior to tubule assays, HUVECs were stained with CellTracker Green CMFDA (Life Technologies, C7025) accordingly to the vendor protocol. The labeled HUVECs were then seeded onto Geltrex^™^ matrices (1x10^5^ cells/well) in serum-free F-12K media containing 50 ng/mL of rVEGF, or 25 nM of either QK or E7-QK. After a 6 hr incubation, tubule formation was captured from at least 3 random fields/well at 10x magnification using the EVOS FL Auto Cell Imaging System. Network branches and nodes were counted from the collected images to quantify angiogenic network formation.

### Activation of signaling cascades in endothelial cells exposed to peptides presented in solution

HUVECs were incubated in serum-free F-12K media containing 25 nM of QK or E7-QK peptide for 10 min. The cells were then lysed in RIPA buffer (ThermoFisher Scientific, 89901) supplemented with 1% protease and phosphatase inhibitors (Sigma). Protein concentration was quantified through BCA analysis (ThermoFisher Scientific, 23209). Protein samples were resolved by SDS-PAGE and transferred to polyvinylidene difluoride membranes (Immobilon-P, Millipore) overnight at 4°C. Membranes were placed in a blocking solution of 5% non-fat dry milk in TBS containing 0.1% Tween 20 (TBST) for 1 hr at 37°C. Blots were probed with primary antibodies specific for either p-Akt (S473, Cell Signaling, 4060), total Akt (Cell Signaling, 4691S), p-ERK 1/2 (T202/Y204, Cell Signaling 4370L), or total ERK 1/2 (Cell Signaling, 9102S) followed by incubation with HRP-linked secondary antibodies (Cell Signaling, 7074S). Blots were also probed with anti-β-tubulin (Abcam, ab21058) to ensure even loading of protein lysates. Proteins were detected by enhanced chemiluminescence using Clarity Western ECL substrate (BioRad, 170–5060). Densitometric analyses of immunoblots were performed using ImageJ, and the Densitometric Units (DU) measured for the phosphorylated signaling molecule were normalized to the DUs obtained for the respective total amount of protein.

### Activation of signaling cascades in cells seeded onto peptide-coated HA disks

HA disks were placed within individual wells of a 24 well plate and then coated for 2 hrs with 0.5 mL of TBS containing 25 nM of either QK or E7-QK peptide. As a negative control, disks were incubated for 2 hr with TBS (“uncoated”). After this interval, the disks were washed briefly with TBS to remove unbound peptide. 5.0x10^5^ HUVECs were seeded onto the HA disks and allowed to attach for 30 min at 37°C. The 30 min time point was selected because of the requirement for cells to adhere to the disks. To monitor ERK and Akt signaling in cells interacting with peptide-bound disks, it was necessary to first remove cells that had not attached to the disks (as these cells would lack ERK/Akt activation). Pilot studies were conducted (not shown) to identify the shortest time interval, 30 min, that would allow strong enough cell adhesion to the disks to withstand the wash steps needed to remove the unbound cells. After the 30 min binding interval, unbound cells were removed by several washes in TBS, and then the disks with adherent HUVECs were submerged in RIPA buffer containing 1% protease and phosphatase inhibitors for 20 min at 4°C to lyse the attached cells. Cell lysates were concentrated using Amicon Ultra-0.5 Centrifugal Filter Devices (Millipore, UFC500396) and protein concentration was quantified by BCA analysis. Protein samples were resolved by SDS-PAGE and transferred to polyvinylidene difluoride membranes overnight at 4°C. Membranes were probed for p-Akt, total Akt, p-ERK 1/2, total ERK 1/2 and β-tubulin as described previously.

### Statistical analysis

Peptide/graft binding experiments were performed three independent times, with each experiment performed in duplicate. Migration assays and endothelial tubule formation assays were conducted in three independent experiments, each experiment executed with triplicate wells. A student’s t-test was used to measure the differences between experimental groups. Values were considered significant with a P value of <0.05. Immunoblots shown are representative of at least three independent experiments. Densitometric analysis comparing the relative phosphorylated protein levels versus total levels were averaged between the independent experiments. Relative densitometric values were considered significant with a P value of <0.05.

## Results

### E7-QK exhibits better binding to bone graft materials than QK

**Fig 1:**
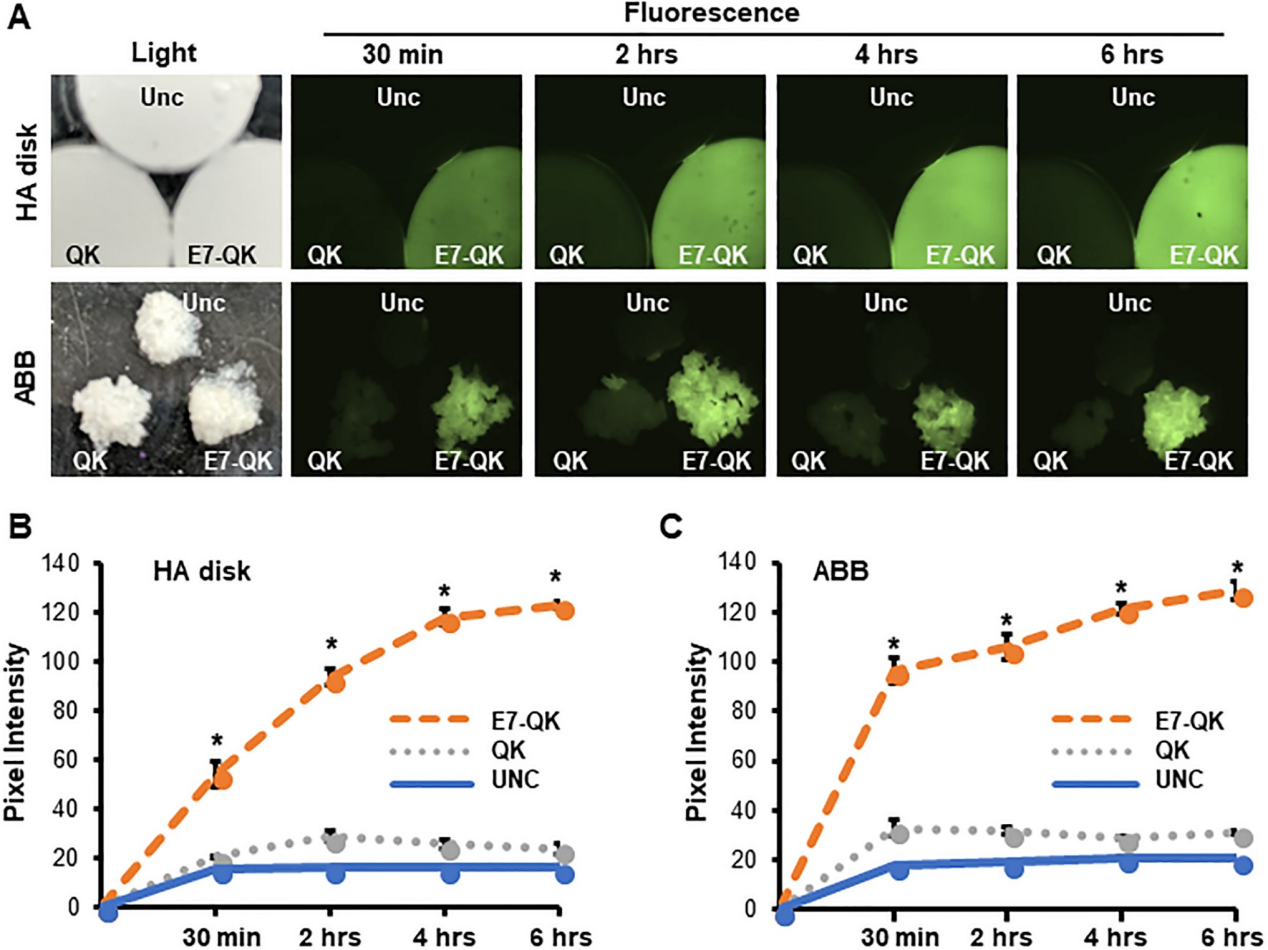
E7 domain directs greater loading of E7-QK onto HA disks and ABB particles. (A) HA disks or ABB particles were incubated with 1 μM of FITC-tagged QK or E7-QK peptides for time points ranging from 30 min to 6 hrs. As a control, samples were incubated in TBS (uncoated, “Unc”). Following these incubations, samples were washed in TBS and imaged by fluorescent microscopy, which revealed greater binding of E7-QK. Unc, QK and E7-QK coated samples were imaged within the same field (as depicted in the light microscopy image) to enable a direct comparison. (B &C) Images from HA disks (B) or ABB (C) were analyzed by Image J to quantify fluorescence intensity. Values represent means and S.E.s from three independent experiments. * denotes p<0.05 (relative to Unc samples).

FITC-labeled QK or E7-QK peptides were coated onto HA disks or ABB particles for time intervals ranging from 30 min to 6 hrs. Fluorescent images of the QK and E7-QK coated materials (as well as uncoated control materials, Unc) were captured at various timepoints to monitor the changes in peptide binding over time. As shown in Fig 1A, a greater amount of E7-QK was apparent on both HA and ABB substrates at all time points when compared to grafts coated with QK peptide or uncoated grafts. To quantify peptide binding to the substrates, images were examined for pixel intensity using Image J (Fig 1B & 1C). The pixel intensities confirmed that there was a substantial increase in the amount of bound E7-QK relative to QK. Furthermore, the amount of E7-QK that bound to HA and ABB increased over the 6 hr interval, whereas maximal binding of the QK peptide was observed within ~30 min. Interestingly, E7-QK appeared to bind more rapidly to ABB than HA disks, achieving ~70% maximal binding within 30 min. The reason for this discrepancy is currently unclear, but may relate to the porous microarchitecture of the ABB particles. It is possible that ABB offers a larger surface area for peptide binding. Nonetheless, despite differences in the early kinetics of peptide binding to ABB vs. HA disks, these data clearly show that the E7 domain is effective in improving the coupling of QK to two distinct graft materials.

### E7-QK elicits a proangiogenic phenotype in endothelial cells

**Fig 2:**
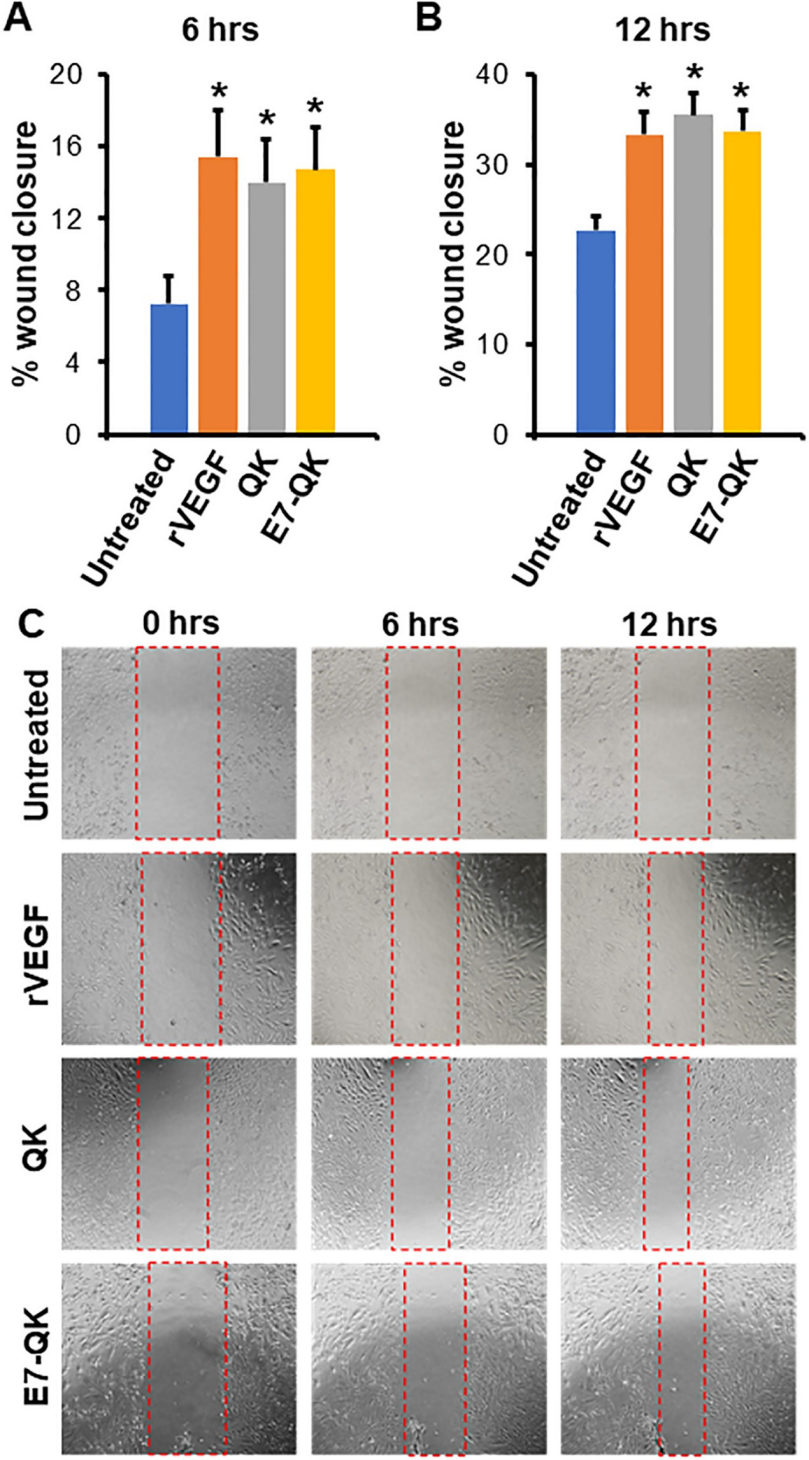
-QK stimulates endothelial cell migration. E7 Scratch wounds were introduced into HUVEC monolayers, and then cells were incubated with serum-free media (untreated) or serum-free media containing 50 ng/ml rVEGF, or 25 nM of either QK or E7-QK. (A&B) Analyses of cell migration at 6 (A) and 12 (B) hrs indicated that all of the treatments elicited more robust migration as compared with controls. (C) Representative images with defect area indicated by red dashed lines. Values represent means and S.E.s from 3 independent experiments. * denotes p<0.05.

Having verified that the E7 domain improved peptide binding to graft, the effect of E7-QK on endothelial cell behavior was next evaluated. While prior studies have confirmed the angiogenic properties of the QK peptide [35, 37, 38], it was important to insure that the addition of the E7 domain did not negatively impact QK’s activity. To assess endothelial cell migration in response to E7-QK, a scratch wound assay was performed. Linear scratch defects were created in confluent HUVEC monolayers, and then cells were incubated with serum-free media (“untreated”) or serum-free media containing either rVEGF (positive control), QK, or E7-QK. Cell migration into the scratch wound was monitored in real-time using the EVOS imaging system, and changes in scratch wound width were quantified at 6 and 12 hrs (Fig 2A and 2B, respectively). At both time points, HUVECs treated with rVEGF, QK, or E7-QK exhibited greater migration as compared with HUVECs in control media (representative images in Fig 2C).

**Fig 3:**
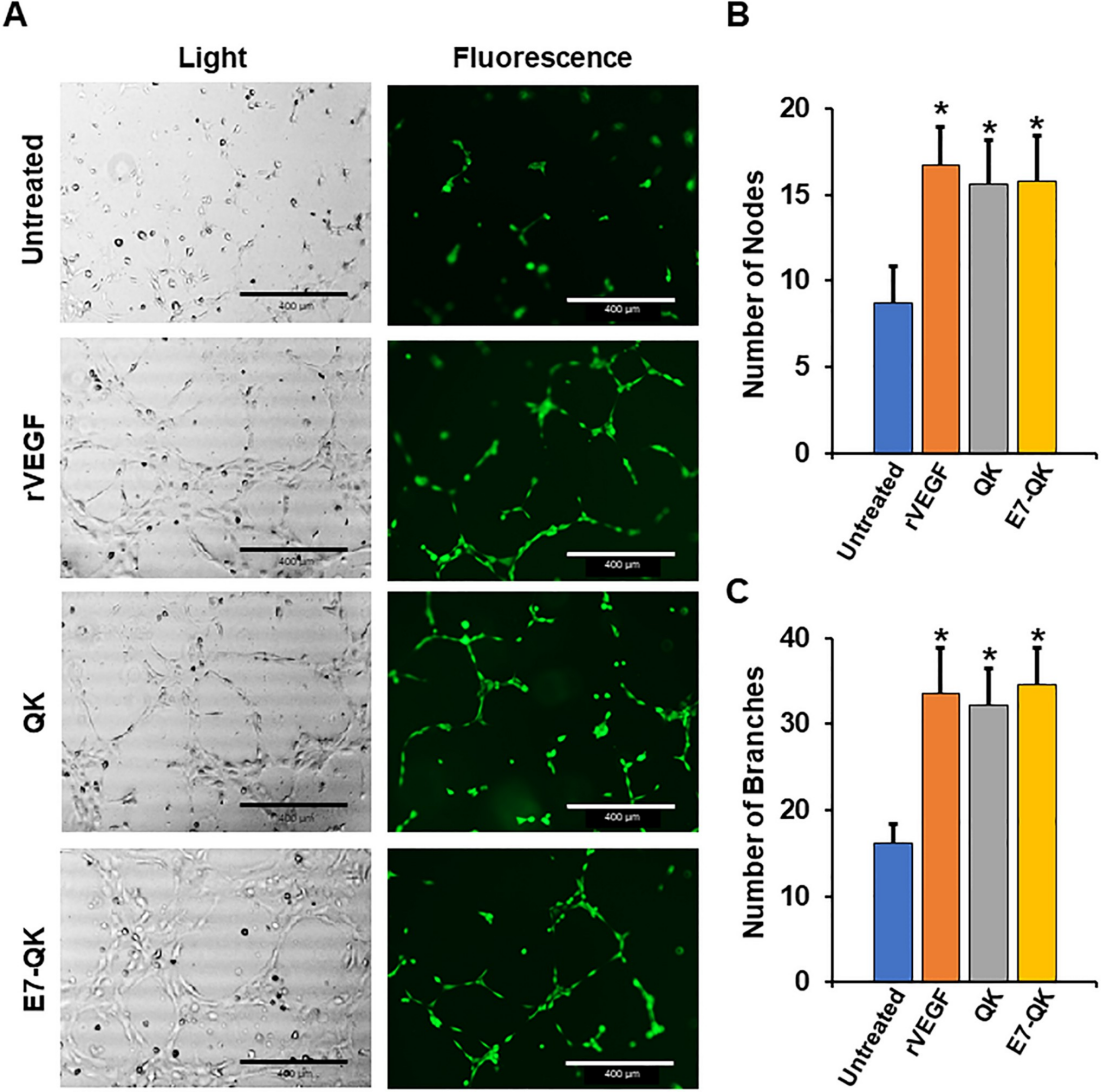
E7-QK induces endothelial tubule formation. HUVECs were pre-labelled with Cell Tracker Green dye, and then seeded onto GELTREX matrices in either serum-free media (untreated) or serum-free media containing 50 ng/mL rVEGF, or 25 nM of either QK or E7-QK peptides. (A) Tubule formation was monitored at 6 hr after cell seeding (phase contrast images in left panels; fluorescent images in right panels). Greater nodal formation (B) and branching (C) were observed in all experimental groups relative to controls. Images are representative of 3 random fields/experimental well. Values represent means and S.E.s from 3 independent experiments, with each experiment performed in triplicate. Scale bar = 400μm.

To further evaluate E7-QK activity, an endothelial tubule formation assay was performed. HUVECs were seeded onto GELTREX matrices, and then cells were incubated for 6 hrs with serum-free media (untreated), or serum-free media containing rVEGF, QK, or E7-QK. Images taken at the end of this interval showed a high volume of interconnectivity between HUVECs grown in E7-QK, rVEGF, or QK solutions (Fig 3A). However, in the absence of any angiogenic stimulus, the cells were unable to form tubules. A quantitative analysis of tubule nodes (Fig 3B) and branches (Fig 3C) revealed no differences in the capacity of E7-QK, rVEGF and QK to stimulate tubule formation, although all three stimuli induced significantly more tubule formation than the untreated control. These studies, combined with the cell migration assays, confirmed that the addition of the E7 domain did not diminish the potency of the QK peptide in stimulating angiogenic endothelial cell behaviors.

### E7-QK activates angiogenesis-associated signaling cascades

**Fig 4:**
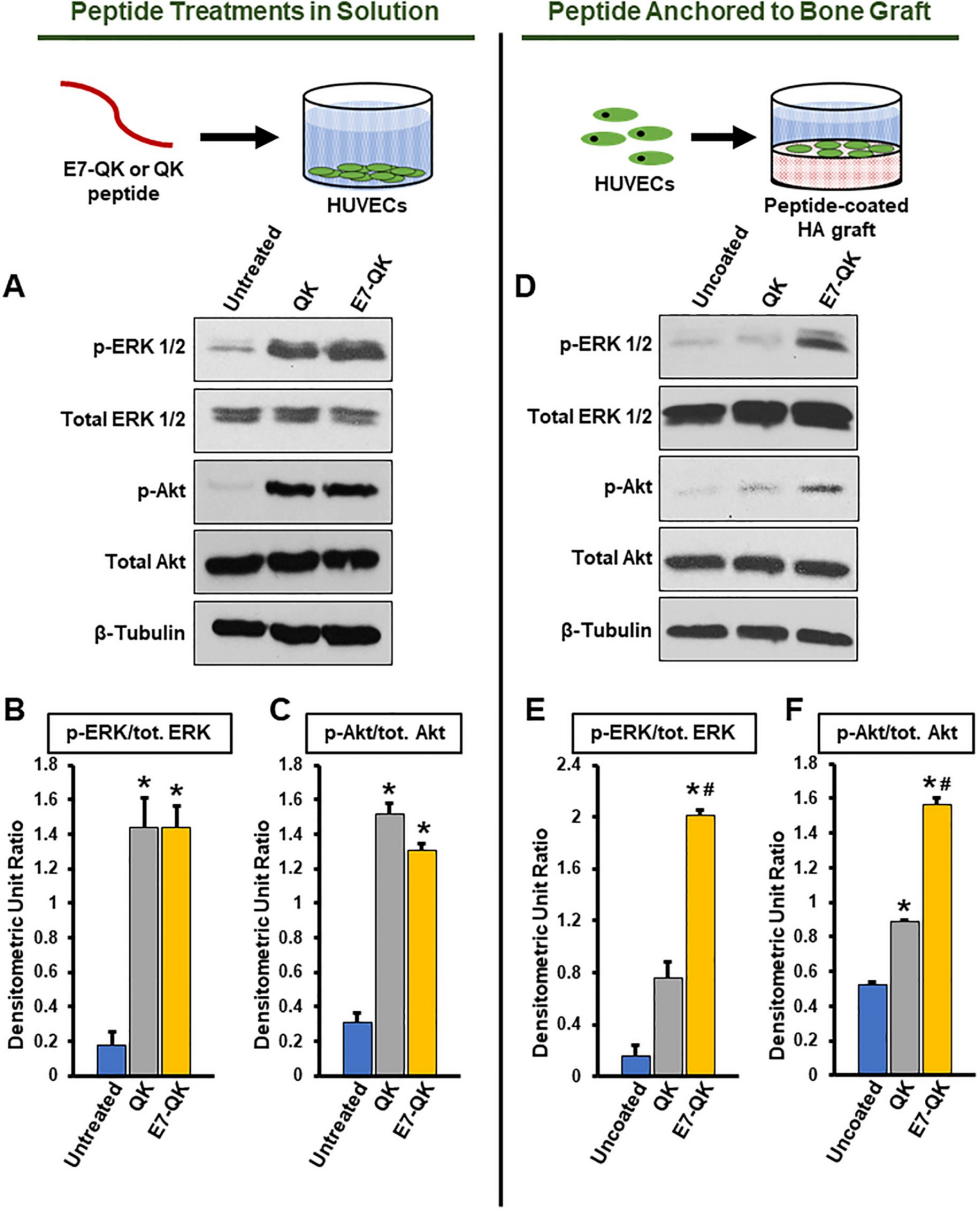
Cell signaling activation in cells exposed to E7-QK in solution, or E7-QK immobilized on HA disks. (A) HUVECs were incubated for 10 min with either serum-free media (untreated) or serum-free media containing 25 nM of QK or E7-QK peptide. Cells were then lysed and immunoblotted for p-ERK1/2, total ERK 1/2, p-Akt, total Akt, or β-tubulin. (B&C) Densitometric analyses of blots were conducted using Image J, and values for phosphorylated ERK 1/2 (B) and Akt (C) were compared to densitometric values for total ERK 1/2 and Akt (Densitometric Unit Ratio). Graphs depict means and S.E.s from three independent experiments. * denotes p<0.05 (relative to Untreated samples). (D) HA disks were coated for 2 hrs with either TBS (uncoated) or TBS containing 25 nM of QK or E7-QK peptides. Disks were then washed to remove unbound peptides. HUVECs were seeded onto the treated disks and allowed to adhere for 30 min. The cells were lysed, and after a concentration step, the lysates were immunoblotted for p-ERK 1/2, total ERK 1/2, p-Akt, total Akt and β-tubulin. (E&F) Densitometric analyses of blots were conducted using Image J, and values for phosphorylated ERK 1/2 (E) and Akt (F) were normalized to total protein levels. Graphs depict means and S.E.s from three independent experiments. * denotes p<0.05 (relative to Uncoated samples) and # denotes p<0.05 (relative to QK peptide coated disks).

The angiogenic activity of E7-QK was also evaluated by monitoring intracellular signaling cascades downstream of VEGFR2 activation, specifically, the phosphorylation of ERK and Akt kinases [41, 42]. HUVECs were treated for 10 min with either serum-free media or serum-free media containing QK or E7-QK. Cells were then lysed and immunoblotted for phosphorylated and total levels of ERK 1/2 and Akt. Cells incubated in QK and E7-QK solutions displayed higher levels of p-ERK 1/2 and p-Akt as compared with untreated HUVECs (Fig 4A). Importantly, QK and E7-QK stimulated equivalent activation of ERK 1/2 (Fig 4B) and Akt (Fig 4C), suggesting that the E7 domain did not impair the ability of the QK sequence to bind and activate VEGF receptors.

### Activation of ERK and Akt is increased in cells exposed to HA disks coated with E7-QK versus QK peptides

We next tested whether E7-QK retained its bioactivity when bound to graft materials. To this end, HA disks were coated for 2 hrs with solutions containing either QK or E7-QK, or incubated in saline (uncoated) as a control. A 2 hr time point was selected to model the clinical setting, in which graft particles would be coated with the peptides just before graft placement. At 2 hrs, peptide binding to ABB is near maximal (Fig 1C). Following peptide coating, the disks were washed to remove unbound peptides. HUVECs were seeded onto the treated disks for 30 min to allow cell interaction with the peptide-coated surfaces. Cells were exposed to the disks for 30 min (rather than the 10 min time point used in Fig 4A) because of the time required for cells to first adhere to the graft material, and then respond to the E7-QK or QK bound to the disk surface. After the binding interval, disks were washed to remove unbound cells, and then adherent cells were lysed and immunoblotted for activation of ERK 1/2 and Akt (Fig 4D). Densitometric analysis of phosphorylated protein levels normalized to total levels revealed strikingly higher levels of p-ERK 1/2 (Fig 4E) and p-Akt (Fig 4F) in cells attached to QK-coated, or uncoated, disks. Taken together, these data suggest that the E7 domain can be used to concentrate active QK peptides onto bone graft materials.

## Discussion

The functionalization of non-autologous bone graft materials with bioactive factors constitutes a highly active area of research. Both osteoinductive and angiogenic factors have been investigated for their potential to improve graft performance, however better methods are needed for coupling these factors to the graft surface [43–46]. In the current study we evaluated a method for increasing the binding of an angiogenic peptide, QK, to the surface of calcium phosphate materials. By adding an E7 domain to the QK peptide, we achieved a 4-6-fold enrichment in the amount of peptide loaded onto two graft materials used in the clinic, ABB and synthetic HA. Similar results were reported by Lee et al., who showed that QK binding to HA biomaterials could be enhanced by adding an HA-binding sequence derived from osteocalcin [43]. In tandem with HA binding domains, the QK peptide has been engineered with sequences that have affinity for other bone matrix molecules such as collagen I [47]. As an alternative to peptides with matrix binding domains, soluble QK peptides have been encapsulated within hydrogels [39, 48, 49]. Upon implantation, QK either diffuses from the hydrogel, or is released as the hydrogel degrades. While peptide-containing hydrogels have many worthwhile features, bone grafting procedures often require the use of mineralized materials, which have greater mechanical strength, and offer architectural and biochemical properties reflective of native bone [50, 51].

The use of E7-QK to augment the osteoregenerative capacity of graft materials offers several advantages. First, E7-QK peptides can, in theory, be applied to any type of calcium phosphate, providing versatility in clinical applications. While the current investigation focused on ABB and HA, other studies have demonstrated that the E7 domain binds with high affinity to all calcium phosphate materials tested to date including several types of human allograft as well as β-tricalcium phosphate [22, 23]. The E7-QK peptide can be stored as a lyophilized powder and reconstituted in saline for immediate use, suggesting that this coating technique may be readily implemented in the clinic. As another benefit, short, synthetic peptides are simpler, and more cost-effective, to produce than the full-length proteins from they were derived [52]. Recombinant proteins are typically generated via host cell systems, which can introduce contaminants such as cellular by-products or pathogens [53]. In contrast, large amounts of highly pure synthetic peptides can be produced by a commercial peptide synthesizer.

The capacity of the QK peptide to substitute for rVEGF in stimulating neovascularization is well-established. QK stimulates the same endothelial cell behaviors as rVEGF [37], and has comparable angiogenic potency in multiple animal models [37–39]. Consistent with this literature, we find that QK induces endothelial cell migration, tubule formation and activation of key signaling molecules such as ERK and Akt. Importantly, these functions of QK are not diminished by the addition of the E7 domain. E7-QK retains full activity when presented to cells either in solution, or following immobilization onto HA disks. In fact, because significantly more E7-QK than QK binds to HA, endothelial cells seeded onto E7-QK-coated HA disks are strongly activated, as evidenced by ERK 1/2 and Akt phosphorylation. Contrarily, cells exposed to QK-coated HA disks display limited activation of ERK 1/2 and Akt, consistent with the poor binding of QK to HA. The fundamental concept that concentrating QK onto a material surface can enhance endothelial cell activation is supported by other studies. For example, Yang et al. covalently linked QK to electrospun scaffolds, and found that endothelial cells adherent to the QK-conjugated scaffolds had greater viability than cells attached to scaffolds with passively adsorbed QK [54].

In summary, our collective results suggest that the E7 domain serves as an effective tool for concentrating angiogenic peptides on the surface of diverse calcium phosphate graft materials. This, in turn, should enable higher doses of the peptide to be delivered within the graft site, providing a more robust angiogenic stimulus. Given that inadequate, or delayed, vascularization is a major impediment to bone regeneration, the current study offers a promising new therapeutic modality for enhancing the performance of non-autologous graft materials commonly used in craniofacial and orthopedic procedures.

## Supporting information

S1 Fig Original western blot images from peptide treatments in solution. (A) p-ERK 1/2. (B) p-Akt. (C) total ERK 1/2. (D) total Akt. (E) β-Tubulin.
(TIF)

S2 Fig Original western blot images from peptide anchored to graft. (A) p-ERK 1/2. (B) p-Akt. (C) total ERK 1/2. (D) total Akt. (E) β-Tubulin.
(TIF)
