# Supplementary figures and images for: The addition of a polyglutamate domain to the angiogenic QK peptide improves peptide coupling to bone graft materials leading to enhanced endothelial cell activation
Source: PLoS One. 2019 Mar 11;14(3):e0213592. doi: 10.1371/journal.pone.0213592 (PMC6411101; doi:10.1371/journal.pone.0213592)

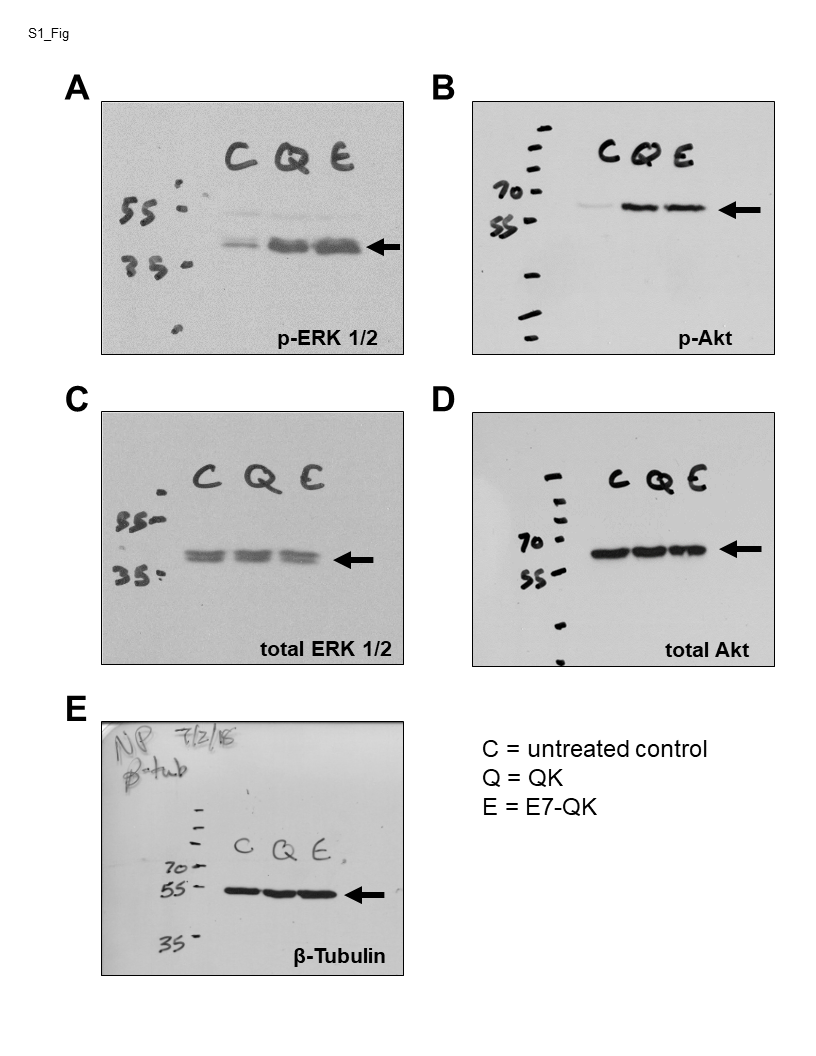

Supplement: S1 Fig — (A) p-ERK 1/2. (B) p-Akt. (C) total ERK 1/2. (D) total Akt. (E) β-Tubulin. (TIF) [file pone.0213592.s001.tif]

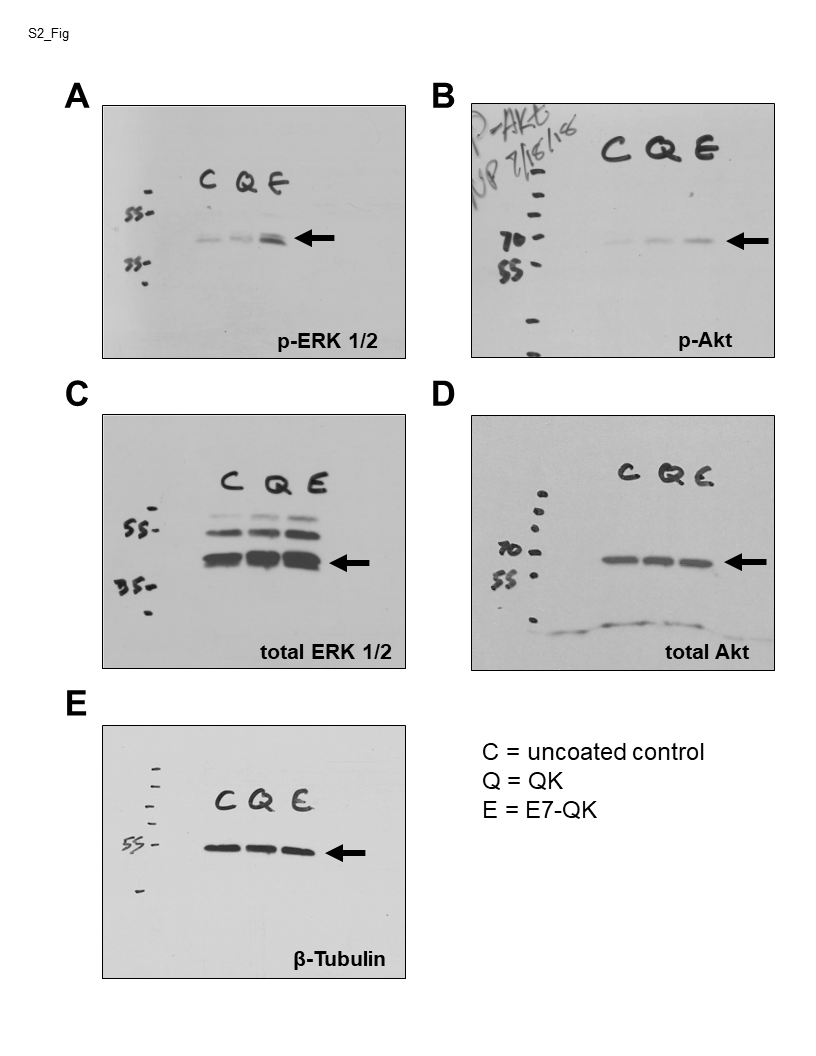

Supplement: S2 Fig — (A) p-ERK 1/2. (B) p-Akt. (C) total ERK 1/2. (D) total Akt. (E) β-Tubulin. (TIF) [file pone.0213592.s002.tif]
